# The Effect of MicroRNA *bantam* on Baculovirus AcMNPV Infection *in Vitro* and *in Vivo*

**DOI:** 10.3390/v8050136

**Published:** 2016-05-16

**Authors:** Xiaojie Shi, Zihan Ran, Sisi Li, Juan Yin, Jiang Zhong

**Affiliations:** State Key Laboratory of Genetic Engineering, Department of Microbiology and Microbial Engineering, School of Life Sciences, Fudan University, Shanghai 200438, China; xiaojie.shi@hotmail.com (X.S.); 15110700014@fudan.edu.cn (Z.R.); liss6@sustc.edu.cn (S.L.); yinjuan@fudan.edu.cn (J.Y.)

**Keywords:** microRNA, *bantam*, baculovirus, virus-host interaction, biopesticide

## Abstract

The role of microRNA *bantam*, one of the most abundant microRNAs in Sf9 cells, was studied for its role in baculovirus infection *in vitro* and *in vivo*. The expression level of *bantam* was increased after AcMNPV infection in Sf9 cells and in *Spodoptera litura* larvae. In Sf9 cells, application of *bantam* inhibitor or mimic altered the expression of many virus genes, the most affected gene being *lef8*, *gp41* and *p10*, the expression level of which was increased by 8, 10 and 40 times, respectively, in the presence of *bantam* inhibitor. Virus DNA replication was decreased in the presence of *bantam* mimic and increased in the presence of bantam inhibitor in a dose dependent manner. However, the production of budded virus did not change significantly. Feeding the larvae of *S. litura* and *Spodoptera exigua* with *bantam* antagomiR, a more stable form of the inhibitor, resulted in an abnormal larval growth and a decreased pupation rate. In *S. litura*, larvae died 3.5 days sooner than the control when *bantam* antagomiR was applied, together with AcMNPV. In infected *S. exigua*, larval mortality increased from 47% without antagomiR to 80% with it. The results suggest that microRNA *bantam* plays an important role in insect growth, as well as in baculovirus-insect interaction.

## 1. Introduction

MicroRNA is a kind of highly conserved endogenous small RNA that regulates gene expression at the post-transcriptional stage. They have major roles in cell proliferation, apoptosis, development, metabolism, and many other biological processes in almost all eukaryotic organisms [1]. MicroRNA is also known to play important roles in virus–host cell interactions [2,3]. Changes in cellular microRNA pattern in virus-infected cells have been reported for different viruses [4,5,6]. Some host cell microRNAs interfere with virus infection [7,8,9], while some others are exploited by viruses to facilitate their own replication [10]. Viruses may also encode their own microRNA to interfere with host defense mechanism, or to regulate cellular and virus gene expression [11,12,13].

Baculovirus is a group of invertebrate virus, widely used in the production of recombinant proteins in insect cells. It is also a potential biological pesticide. Mehrabadi *et al.* [14] studied the microRNAome of Sf9 cells before and after the infection of baculovirus *Autographa californica* multicapsid nucleopolyhedrovirus (AcMNPV) by a combination of deep sequencing, bioinformatics, and experimental approaches. They identified 90 novel and conserved microRNAs, with 76 identical to known *Bombyx mori* microRNAs. Similar study by our group also found a few dozen potentially novel microRNA in Sf9 cells. Both studies showed that microRNA *bantam* was one of the most abundant in the cells.

Previous studies showed that microRNA *bantam* was a versatile player in many biological processes in *Drosophila*. As an essential component in Hippo, Notch, and epidermal growth factor receptor (EGFR) pathways [15,16,17], it controls cell proliferation and apoptosis in many cell types [18,19,20] and fly development [21,22], possibly by targeting the proapoptotic gene *hid* [23]. It also regulates the production of the molting hormone ecdysone [24] and even directly targeting *Clk* gene, which is responsible for the maintenance of circadian rhythms [25]. However, the role of microRNA *bantam* in lepidopteran insects has not been well studied yet.

Here, we studied the role of microRNAs *bantam* in AcMNPV infection. We found that *bantam* level was increased after virus infection both in Sf9 cells and in *Spodoptera* larvae, and altering *bantam* levels by mimic and inhibitors affected the pattern of virus gene expression, viral DNA replication in Sf9 cells, and affected virus infectivity in the larvae.

## 2. Materials and Methods

### 2.1. Cells, Viruses, MicroRNA Mimic, Inhibitor and AntagomiR

Insect Sf9 cells were maintained at 27 °C using TNM-FH medium (Sigma-Aldrich, St. Louis, MO, USA) supplemented with 10% FBS. Cell viability was determined by CCK8 reagent (TOYOBO, Osaka, Japan) following manufacturer’s instructions. Recombinant baculovirus AcEGFP, which was constructed by inserting *egfp* gene in the *polyhedrin* locus of AcMNPV [26], was used for *in vitro* infection of Sf9 cells. Wild-type AcMNPV (strain 1A) was used for *in vivo* infection of *Spodoptera litura* (leafworm) and *Spodoptera exigua* (beet armyworm) larvae.

*bantam* mimic, inhibitor and antagomiR were chemically synthesized and modified by GenePharma (Shanghai). *bantam* mimic was unmodified RNA oligo with the same sequence as *bantam* (5′-UGAGAUCAUUGUGAAAGCUGAU-3′). *bantam* inhibitor and antagomiR were complementary to *bantam* (5′-AUCAGCUUUCACAAUGAUCUCA-3′) with modifications. Both of them had 2′-*O*-methyl modification in the sugar, while antagomiR had additional modifications of phosphorothioate backbone for first two ribonucleotides at both the 5′ and 3′ ends, and a cholesterol conjugation [27]. As a more stable form of inhibitor, antagomiR was used for *in vivo* experiments. Control RNA oligos for mimic, inhibitor and antagomiR were provided by the manufacturer.

### 2.2. Transfection and Infection

MicroRNA mimic and inhibitor were used for *in vitro* experiments. RNA oligos were dissolved in RNase-free water to the concentration of 20 μM, and used to transfect the overnight culture of Sf9 cells in a 24-well plate using Turbofect for siRNA reagent (Fermentas, Waltham, MA, USA), following manufacturer’s instructions. The culture medium was replaced by fresh medium, and, when necessary, virus was added to the required MOI (multiplicity of infection) for infection. The cells were transfected again to maintain the effective level of mimic or inhibitor if the culturing time was over 3 days.

### 2.3. Real-Time PCR

Total small RNAs (<200 nt) were harvested from Sf9 cells or insect tissues (grinded in liquid nitrogen) using miRVana microRNA isolation kit (Ambion, Life Technologies, Carlsbad, CA, USA). RT-qPCR detection of microRNAs was performed using miScript (Qiagen, Venlo, The Netherlands) according to instructions. To determine the expression level of virus genes, total RNA was extracted from AcEGFP-infected Sf9 cells (MOI = 0.5 pfu/cell) at different time points for early, late, and very late genes, by TRIzol, and mRNA was reverse-transcribed to cDNA using Primescript RT Master Mix (TaKaRa, Shiga, Japan). Virus DNA in infected cells was extracted by Cell Total DNA Extraction Kit (Tiangen, Beijing, China). Real-time PCR for virus DNA and cDNA were carried out using iTaq mix (Bio-Rad, Hercules, CA, USA). Primers for real-time PCR are listed in Table 1. Stratagene MX3000p was employed for quantitative PCR and data processing was done with software MxPro. The 2^−ΔΔt^ method was used to calculate the relative level of microRNA or mRNA by comparing to the internal controls, which were U6 RNA or *tubulin* mRNA for microRNA and virus mRNA, respectively. All experiments were repeated three or more times, and the representative results were shown.

### 2.4. Insect Experiments

Lavae of *S. litura* and *S. exigua* were reared individually in polymer cups on artificial diet (Keyun Biocontrol, Jiyuan, China), under the condition of 28 °C, 70%–90% humidity, and photoperiod of 15:9. Larvae of the same hatching time were used for experiments.

AntagomirR was used to silence *bantam* in larvae. The *bantam* antagomirR and control antagomiR were dissolved in RNase free water to the concentration of 75 nM. They were added to the surface of a small piece of diet every day so that they could be consumed completely. MicroRNA antagomiRs were administrated on a daily basis from early 2nd instar until the pre-pupal stage. Wild type AcMNPV inclusion bodies (2 × 10^7^ polyhedra/mL) was inoculated *per os* by surface contamination of the diet, one day after antagomiR administration for the first time, and once again 2 days later. There were no less than 15 larvae in each group of treatment. Mortality and pupation were recorded daily.

## 3. Results

### 3.1. bantam Level after Infection

**Figure 1:**
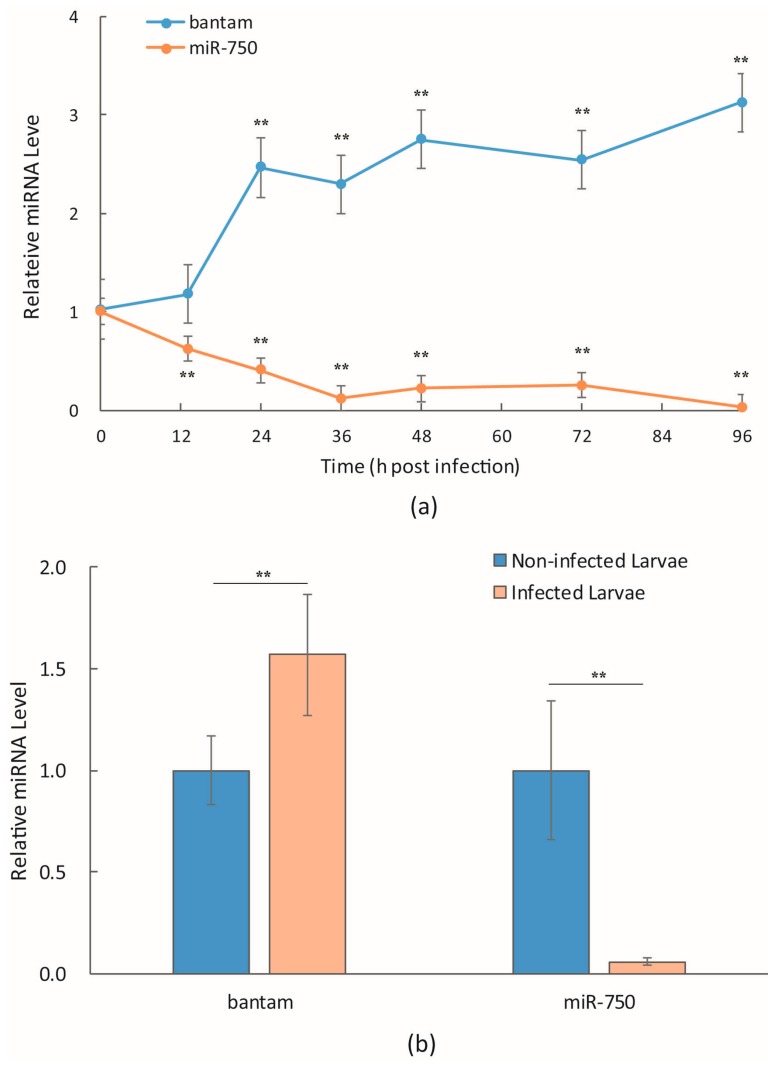
Expression level of microRNA *bantam* in AcMNPV-infected Sf9 cells and *Spodoptera litura* larvae. Total small RNA samples were prepared from infected Sf9 cells at different time points (**a**), or infected *S. litura* larvae (4 d p.i.) (**b**). The levels of microRNA *bantam* and *miR-750* were determined with real-time RT-PCR using U6 RNA as the internal control. The fold change comparing to the non-infected control was shown. The means of three or more replicates were shown, with the error bars indicating the standard deviation. The significance of difference between certain time point and 0 h (**a**), or between infected and non-infected larvae (**b**), was also shown (*: *p* < 0.05; **: *p* < 0.01).

Mehrabadi *et al.* [14] and our own unpublished data on microRNAome of Sf9 cells showed that *bantam* was one of the most abundant microRNAs, which accounted for more than 5% of total microRNA. Our sequence data also suggested that the level of *bantam* increased late in AcMNPV infection. This was confirmed with real-time quantitative PCR in infected Sf9 cells and *S. litura* larvae (Figure 1). Unlike most microRNAs that decreased in the expression level after infection, as represented by *miR-750* in Figure 1, the level of *bantam* increased to about 2.5-fold of uninfected cells 24 h p.i., and remained at a high level late in the infection (Figure 1a). The level of *bantam* was also about 1.5-fold higher than the control in AcMNPV-infected *S. litura* larvae (Figure 1b).

### 3.2. The Effect of bantam Mimic and Inhibitor on AcMNPV Replication in Sf9 Cells

**Figure 2:**
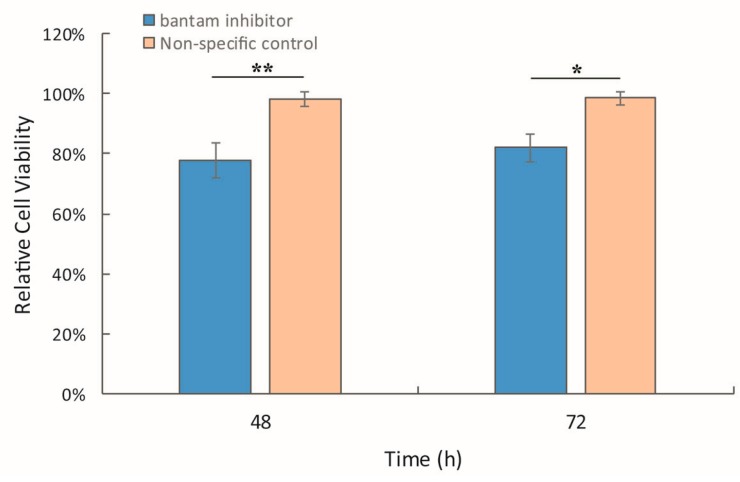
The effect of *bantam* inhibitor on Sf9 cells. Sf9 cells were transfected with *bantam* inhibitor (200 nM), and cell viability was determined 48 h and 72 h post treatment. The cell viability in blank control was set as 100%. The means of three replicates was shown, with the error bars indicating the standard deviation. The significance of difference between *bantam* inhibitor and non-specific treatment was also shown (*: *p* < 0.05; **: *p* < 0.01).

The *bantam* mimic and inhibitor were used to modify the *bantam* level in Sf9 cells in order to study the effect of *bantam* in AcMNPV infection. In the presence of *bantam* inhibitor (200 nM), the viability of Sf9 cells was about 20% lower than the blank control (Figure 2). Treatment with non-specific inhibitor control had no effect on cell viability.

**Figure 3:**
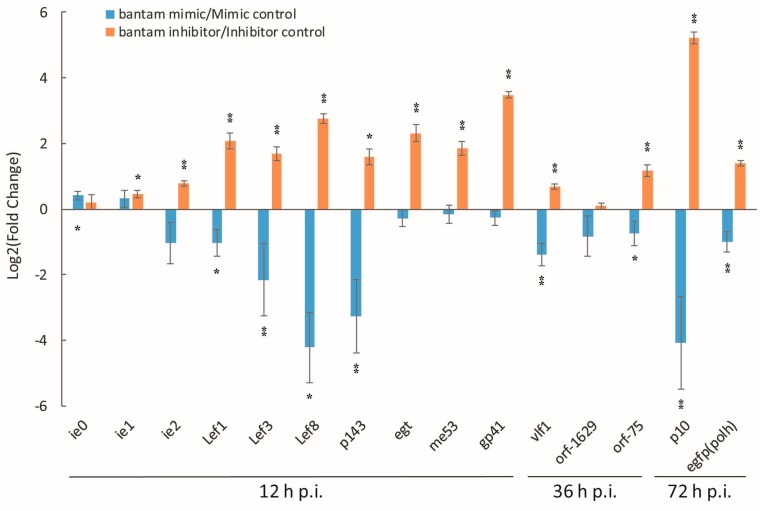
The effect of *bantam* mimic and inhibitor on the transcription of virus genes in Sf9 cells. Total mRNA was prepared from AcEGFP-infected Sf9 cells in the presence of *bantam* inhibitor (200 nM) or mimic (400 nM), and the transcription level of representative virus genes was determined with real-time RT-PCR. Cellular *tubulin* gene was used as the internal control. Sampling time was shown under the gene names. The data was normalized with either inhibitor control or mimic control. The mean of three or more replicates was shown, with the error bars indicating the standard deviation. The significance of difference between *bantam* mimic and mimic control, or between *bantam* inhibitor and inhibitor control was also shown (*: *p* < 0.05; **: *p* < 0.01).

AcEGFP, a recombinant AcMNPV with *egfp* gene in the *polyhedrin* locus, was used to infect Sf9 cells in the presence of *bantam* mimic or inhibitor, and the expression of a series of virus genes was determined with real-time RT-PCR. These genes included virus immediate early and delayed early genes *ie0*, *ie1*, *ie2*, *egt*, *p143*, *me53*, *lef1*, *lef3*, *lef8*, and *vlf1*, late genes *gp41*, *orf1629* and *orf75*, and very late genes *p10*, *polh* (represented by *egfp*). Different time points were used for genes of different categories, which were 12 h, 36 h and 72 h p.i. for immediate early and delayed early, late, and very late genes, respectively. The results showed that *bantam* mimic and inhibitor significantly affected the transcription of many of the tested genes (Figure 3). In most cases, the expression level was higher than the control in the presence of *bantam* inhibitor (the logarithm of fold change was positive), and lower than the control in the presence of *bantam* mimic (the logarithm of fold change was negative). Among all genes, the three most affected genes were *lef8*, *gp41* and *p10*, the expression level of which increased by about 8, 10 and 40 times, respectively, in the presence of *bantam* inhibitor. The expression of immediate early genes *ie0* and *ie1* remained stable in the presence of either *bantam* mimic or inhibitor.

**Figure 4:**
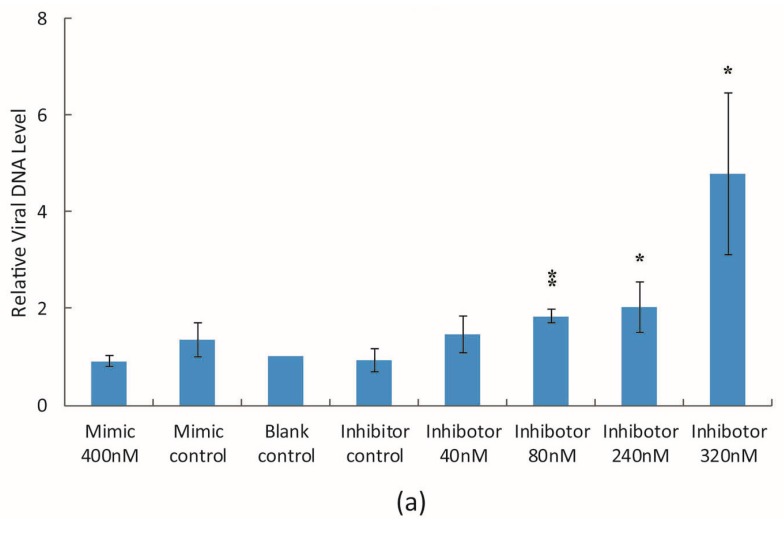

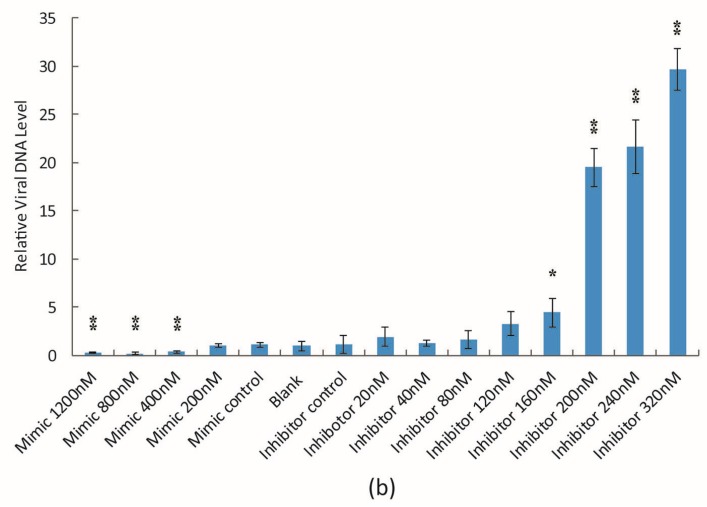
The effect of *bantam* mimic and inhibitor on viral DNA replication in Sf9 cells. Total DNA was prepared from AcEGFP-infected Sf9 cells in the in the presence of *bantam* inhibitor or mimic, and the relative level of viral DNA was determined 48 h p.i. (MOI = 2, (**a**)) or 72 h p.i. (MOI = 0.05, (**b**)) with real-time PCR. *egfp* gene in AcEGFP genome was used as the amplification target, and cellular *tubulin* gene was used as the control. The mean of three or more replicates was shown, with the error bars indicating the standard deviation. The significance of difference between certain concentration of bantam mimic or inhibitor with mimic control or inhibitor control, respectively, was also shown (*: *p* < 0.05; **: *p* < 0.01).

The viral DNA level in infected cells was also determined 48 h p.i. when cells were infected at MOI of 2 pfu/cell, or 72 h p.i. when the cells were infected at MOI of 0.05 pfu/cell, in the presence of different concentrations of *bantam* mimic and inhibitor. A dose-dependent effect was observed in both cases (Figure 4). The more *bantam* inhibitor that was added, the higher the level of virus genome that was detected; the more *bantam* mimic that was added, the lower the level of virus genome that was detected. The effect was more significant at a low MOI. However, *bantam* mimic or inhibitor had no major effect on the titer of budded virus in the medium 48 h p.i. and 72 h p.i..

### 3.3. The Effect of bantam antagomiR in AcMNPV Infection in Spodoptera larvae

**Figure 5:**
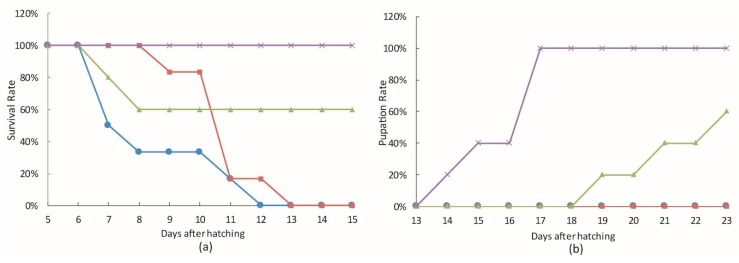

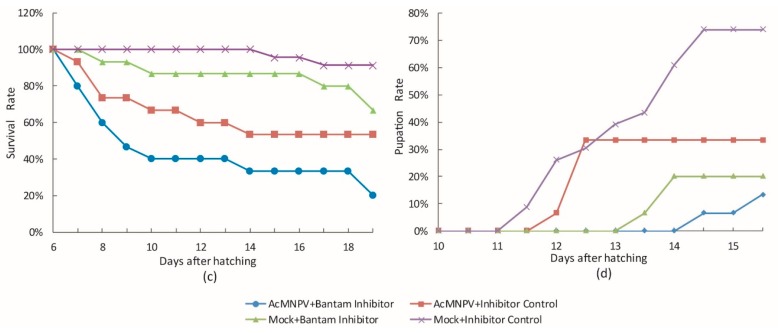
The effect of *bantam*-antagomiR on AcMNPV infection in larvae. Second instar larvae of *S. litura* (**a**,**b**) and *S. exigua* (**c**,**d**) were administrated with *bantam*-antagomiR or control on a daily basis. They were then inoculated with AcMNPV polyhedra *per os*, or mock infected twice, on Day 2 and 4 after first antagomirR administration. Larval death (**a**,**c**) and pupation (**b**,**d**) were recorded. Each group includes 15 or more larvae.

*bantam* antagomiR was used to study the effect of *bantam* on AcMNPV infection *in vivo*. Second instar larvae of *S. litura* and *S. exigua* were treated with *bantam* antagomiR before being inoculated with occlusion bodies of wild type AcMNPV *per os*. As shown in Figure 5, treating larvae with *bantam* antagomiR resulted in a 20% or 35% of larval death in *S. litura* and *S. exigua*, respectively, and a delay in the pupation of survived larvae.

Meanwhile, *bantam* antagomiR also displayed the ability to improve killing by AcMNPV in these insects (Figure 5). In *S. litura*, although 100% mortality was reached either with or without antagomiR treatment, the larvae died sooner with antagomiR treatment than without it. The antagomiR treatment group reached 50% mortality about 3.5 days earlier than the control group. In *S. exigua*, the mortality increased from 47% in the control to 80% in the antagomiR treatment group, and the average time to death was more than 2 days shorter with antagomiR treatment than without it.

## 4. Discussion

RNA interference pathway is thought to be important in virus-host interactions in insect. Exogenous viral RNA could be processed by this pathway to generate antiviral activity [28,29,30], whereas host-encoded microRNA is also produced by this pathway. As a kind of endogenous functional small RNA, host microRNA has important roles in many physiological processes as well as in the defense against infection [31]. Published work [14] and our early data showed that microRNA *bantam* was among the few most abundant microRNAs in Sf9 cells. *bantam* is an insect-specific microRNA identified in *Drosophila*, *B. mori* [32], and some other insects [33,34]. It has also been reported in blood fluke *Schistosoma japonica* [35,36]. Functional study in *Drosophila* [18,19,20,21,22,23,24,25] and *Schistosoma* [36] showed that it plays an important role in development and cell proliferation. In silkworm *B. mori*, *bantam* was found to be constantly expressed in the body wall, silk glands and midgut, but much less abundant in the fat body [32]. However, little is known of its role in lepidopteran insects. The results in the current work suggested that *bantam* also played a similarly important role in *Spodoptera* insects. Inhibition of the *bantam* activity resulted in decreased cell viability in Sf9 cells, and abnormal larvae growth in *S. litura* and *S. exigua*.

Most of the host microRNAs decreased in the expression level after AcMNPV infection. *bantam* was among the few microRNAs of which the expression level increased after infection both in Sf9 cells and in *S. litura* larvae. Similar to the expression of most proteins, the biogenesis of microRNA is under fine regulation. Therefore, the changed *bantam* expression level upon virus infection implied a possible role in virus–host interaction. The expression regulation of *bantam* has not been well studied yet. Since *bantam* is an effector in Hippo, Notch and EGFR/mitogen-activated protein kinase (MAPK) pathways, it is possible that baculovirus infection activated some elements in these pathways, and indirectly increased the expression level of *bantam*.

It is not clear how important the increase in *bantam* level was for the cellular defense against virus infection, since the cells and insects were susceptible to AcMNPV infection, and the production of progeny virus in infected Sf9 cells did not change much in the presence of *bantam* inhibitor. However, altering *bantam* level using a mimic or inhibitor did affect the expression of some important virus genes and the replication of virus DNA. Meanwhile, some other essential genes, such as *ie0* and *ie1*, were less affected. The mechanism of the difference is not clear. MicroRNA tends to have multiple target sites in the genome. Bioinformatic analysis did not find any potential target of *bantam* in these genes. The only target gene known for *bantam* was the proapoptotic gene *head involution defect* (*hid*) in *Drosophila* [23,37]. Inhibition of *hid* by *bantam* will relieve the suppression on caspase activation, and resulted in apoptosis. Whether this is connected to the change in virus gene expression and DNA replication needs further study.

Inhibiting *bantam* activity had more significant effect on AcMNPV infection in *S. litura* and *S. exigua* larvae. *bantam* antagomiR treatment resulted in increased and accelerated killing in AcMNPV-infected larvae. This might relate to the growth retardation effect of *bantam* antagomiR on larvae. *bantam* is known to have a major effect on cell proliferation and apoptosis, the level molting hormone ecdysone [24], and even the circadian rhythms [25] in insects. Treatment of insects with *bantam* antagomiR alone also resulted in abnormal growth, low pupation rate, and even larval death. It is likely that such treatments made the larvae more vulnerable to infection, and enhanced the killing by virus. Preliminary results in our laboratory showed that administration of juvenile hormone partially compensated the effect of *bantam* antagomiR, which is consistent with the reported role of *bantam* in repressing the release of ecdysone.

In summary, we found that the level of microRNA *bantam* increased after AcMNPV infection *in vitro* and *in vivo*. Interfering *bantam* resulted in the change in virus gene expression, virus DNA replication in Sf9 cells, and increased insect killing in *Spodoptera* larvae. Although further studies are needed to elucidate the mechanisms under these observations; these findings provided a new approach to improve baculovirus as a biological pesticide.

## 5. Conclusions

Inhibiting host microRNA bantam resulted in the change in virus gene expression and virus genomic DNA synthesis in AcMNPV-infected Sf9 cells *in vitro*. It also improved AcMNPV infection of *S. litura* and *S. exigua* larvae, most likely through regulating the growth and development of the larvae. These findings may have applications in biological control of insect pests.

## Figures and Tables

**Table 1:** Sequences of real-time PCR primers.

Target	Type	Sequence (5′–3′)
*bantam*	primer assay	CTTTCTGAGATCATTGTGAAAG
*miR-750*	primer assay	CAGTTGCCAGATCTATCTTTC
*U6* (*B. mori*)	primer assay	AGAGACGATTAGCATGGCCC
*ie-0*	upper primer	CATATTCGTGCGAGGCAACG
	lower primer	CGAGTTGACGCTTGCCAAAA
*ie-1*	upper primer	TTTTAACGCGTCGTACACCA
	lower primer	GTTGACGCTTGCCAAAAAGT
*ie2*	upper primer	AGCGAAGAAAACGTGCAGAT
	lower primer	CTCCGACGCAATGTTATCCT
*lef-1*	upper primer	TTCCGCATATGCAAGATTCA
	lower primer	ACCACATCCACCAGTTCCAT
*lef-3*	upper primer	AACCCCCATGAGCGACTATTTG
	lower primer	ACCGCTGTTGATTTTCTTGTA
*lef-8*	upper primer	AATGGCTACTCTGTGGCGGTAA
	lower primer	TTACCGCCACAGAGTAGCCATT
*egt*	upper primer	ACCGTTTCCAGCGATCAACT
	lower primer	TAGAGGCGGAAACGTTGCTT
*me53*	upper primer	CGCATCTCTCCGCCTAAACA
	lower primer	GCGGGCAAACCTTCAAACTT
*p143*	upper primer	AAGTATTTGCCCGAGGAC
	lower primer	ATTTTGGCGATGTGATAAGT
*p35*	upper primer	ACGACACGGGACTTTACGAG
	lower primer	GTTTTTCGACGCTTCGTTGT
*vlf-1*	upper primer	CGTGCCAATTCCAACGGTTT
	lower primer	CAACTCAGCGTGGACGATCT
*gp41*	upper primer	AGAGTTGGGACAGAGCAACG
	lower primer	GCGCCACCGTTGTAAAACTT
*orf-1629*	upper primer	GTTAGGCACGGGAGAA
	lower primer	CGAAGCAGACGACCTT
*p10*	upper primer	GACGCCGTTACGGAAACTAA
	lower primer	GTCTGGAAGATCCGGAACAA
*egfp*	upper primer	CGTAAACGGCCACAAGTTCAT
	lower primer	GGGTCAGCTTGCCGTAGGT
*orf-75*	upper primer	GCTGGCAGTTGGTATGCTTC
	lower primer	ACTCAGTAGGCGACAGGTTG
*β-tublin* (*B. mori*)	upper primer	TTGCATTGGTACACTGGCGA
	lower primer	ACACCAGGTCGTTCATGTTGC

## Abbreviations

The following abbreviations are used in this manuscript:

MOI	multiplicity of infection
AcMNPV	*Autographa californica* multicapsid nucleopolyhedrovirus
